# Time-Resolved Transcriptomics Provides Insights into Cultivar-Dependent Sucrose Loss During Ambient Storage of Sweet Corn

**DOI:** 10.3390/plants15213413

**Published:** 2026-11-03

**Authors:** Yingjie Zhao, Jingtao Qu, Hongru Liu, Zihao Zhang, Wei Gu, Diansi Yu, Hui Wang, Kai Li, Felix San Vicente Garcia, Hongjian Zheng, Yuan Guan

**Affiliations:** 1 CIMMYT-China Specialty Maize Research Center, Crop Breeding and Cultivation Research Institute, Shanghai Academy of Agricultural Sciences, Shanghai 201403, China; zhaoyingjie@saas.sh.cn (Y.Z.); qujingtao@saas.sh.cn (J.Q.);; 2 Key Laboratory of Germplasm Innovation and Genetic Improvement of Grain and Oil Crops (Co-Construction by Ministry and Province), Ministry of Agriculture and Rural Affairs, Crop Breeding and Cultivation Research Institute, Shanghai Academy of Agricultural Sciences, Shanghai 201403, China; 3 Key Laboratory of Agricultural Genetics and Breeding of Shanghai, Crop Breeding and Cultivation Research Institute, Shanghai Academy of Agricultural Sciences, Shanghai 201403, China; 4 Agri-Food Storage and Processing Engineering Technology Research Center, Crop Breeding and Cultivation Research Institute, Shanghai Academy of Agricultural Sciences, Shanghai 201403, China; 5 International Maize and Wheat Improvement Center (CIMMYT), Texcoco 56237, Mexico

**Keywords:** sweet corn, ambient storage, sucrose metabolism, time-resolved transcriptomics, cell wall invertase

## Abstract

Postharvest sucrose depletion severely limits the organoleptic quality and commercial value of sweet corn during ambient storage. To characterize the temporal transcriptional responses associated with cultivar-dependent sugar loss, ears of two supersweet corn hybrids, ‘Shenketian 811’ (SKT) and ‘Overland’ (AFL), were stored under monitored ambient conditions (24–26 °C) for 7 days. Kernel sugars, total soluble solids, whole-ear respiration rates, and transcriptomic profiles were assessed at five time points. Sucrose concentration declined from 77.9 to 30.9 μg mg^−1^ fresh weight in SKT and from 80.3 to 20.3 μg mg^−1^ fresh weight in AFL. Weighted gene co-expression network analysis (WGCNA), followed by within-cultivar module–trait analysis, identified one module positively and one negatively associated with sucrose concentration in both cultivars. Subsequent gene-level correlation analysis of the 1071 genes in these modules identified 571 non-redundant sucrose-associated genes, which were enriched in carbon metabolism, glycolysis/gluconeogenesis, the citrate cycle, and starch and sucrose metabolism. Pairwise differential-expression analysis further identified 194 genes showing recurrent storage-responsive or cultivar-dependent expression, of which five genes involved in sucrose cleavage or sucrose–starch interconversion were prioritized. Notably, the cell-wall invertase gene *ZmINVCW1* exhibited a sustained AFL-biased expression pattern, which was validated by RT-qPCR, while total cell wall invertase activity was also significantly higher in AFL at multiple sampling points. This time-resolved analysis characterizes recurrent transcriptional responses associated with cultivar-dependent sucrose loss and provides a focused framework for further functional studies of postharvest sucrose retention.

## 1. Introduction

Sweet corn (*Zea mays* L. var. *saccharata*) is harvested at the milk stage, when its tender kernels and high soluble-sugar content contribute to its value as a fresh vegetable [1,2]. Sucrose is a major contributor to kernel sweetness and an important determinant of eating quality [1,3]. After harvest, ears remain metabolically active, while rapid sugar loss and changes in texture and aroma severely reduce marketability [1,4,5]. Both storage temperature and cultivar background influence the rate of sugar loss [6,7]. In three sweet corn cultivars, Olsen et al. observed faster eating-quality deterioration at 7 and 18 °C than at 1 and 4 °C; after 10 days at 18 °C, total sugars had declined by 57–74% [3]. Xiao et al. found that kernels stored at 4 °C retained more sucrose and accumulated less starch than those stored at 28 °C [8]. During 5 days of ambient storage, a slower decline in total soluble sugars was observed in three tropical sweet corn cultivars than in three temperate cultivars [9].

Sucrose synthesis and cleavage involve distinct enzymatic reactions. Sucrose-phosphate synthase (SPS) converts UDP-glucose and fructose-6-phosphate to sucrose-6-phosphate, which sucrose-phosphate phosphatase (SPP) subsequently dephosphorylates to sucrose [10,11]. Sucrose synthase (SUS) catalyzes the reversible conversion of sucrose and UDP into fructose and UDP-glucose. In developing maize endosperm, mutation of the sucrose synthase gene *SH1* reduced enzyme activity by more than 90%, decreased starch accumulation, and increased soluble-sugar content [12]. Invertases, by contrast, irreversibly hydrolyze sucrose to glucose and fructose; cell-wall invertases carry out this reaction in the apoplast [10,13]. The maize *Miniature1* (*Mn1*) gene encodes the cell-wall invertase INCW2, and loss of *Mn1* function reduces endosperm hexose availability and seed mass [14,15]. While these metabolic enzymes are well-characterized during grain development, their temporal regulation during postharvest storage remains a critical factor in quality deterioration.

Postharvest studies have linked sugar retention to broader metabolic responses. An integrated analysis of kernels stored at 4 and 28 °C identified 1993 common differentially expressed genes (DEGs) across three storage times and linked the low-temperature response to flavonoid biosynthesis [16]. In three fresh-maize cultivars stored at 20 °C, Chen et al. associated better sweetness retention with smaller increases in *INV* and *BMY* expression and smaller decreases in *SS4* and *SS5* expression [17]. Zhao et al. reported that slurry-ice precooling followed by storage near 0 °C attenuated sucrose loss and was accompanied by differential expression of transcripts annotated as *β-fructofuranosidase* (*sacA*), *SPS*, *SUS*, and *hexokinase* (*HK*), as well as other genes associated with glycolysis and the tricarboxylic acid cycle [18]. A pulsed-light study at 20 °C likewise used transcriptomic and metabolomic analyses to associate delayed postharvest deterioration with changes in antioxidant, cell-wall, ethylene, and sugar-metabolism pathways [19]. Using a segregating sweet corn population, Ren et al. mapped postharvest total soluble-sugar degradation to intervals on chromosomes 2, 4, and 10 and prioritized *Zm00001eb069070*, a putative carbohydrate-kinase gene [20]. Taken together, studies of cultivar variation, storage treatments, transcriptomics, and genetic mapping indicate that postharvest sugar retention is associated with both genetic background and changes in carbon metabolism. Although precooling and refrigeration delay deterioration, sweet corn may encounter ambient temperatures before cooling or during marketing and retail [1,21]. However, comprehensive time-resolved transcriptomic analyses detailing how different genetic backgrounds modulate these complex carbon-metabolism networks during ambient exposure are still limited. Cultivar-dependent transcriptional responses during ambient storage, and their relationship with sucrose loss, warrant further investigation.

To address this gap, we compared postharvest sugar dynamics and transcriptional responses between two supersweet corn hybrids, ‘Shenketian 811’ (SKT) and ‘Overland’ (AFL), during ambient storage for 7 days. Time-resolved sugar measurements and transcriptome profiling were integrated with weighted gene co-expression network analysis (WGCNA) to identify co-expression modules, carbon-metabolism pathways and candidate genes associated with cultivar-dependent sucrose loss. These analyses highlighted candidate regulators, including a cell-wall invertase gene, and provide a framework for functional studies of sucrose retention during ambient storage.

## 2. Results

### 2.1. Changes in Soluble Sugar Content and Physiological Traits During Ambient Storage

**Figure 1:**
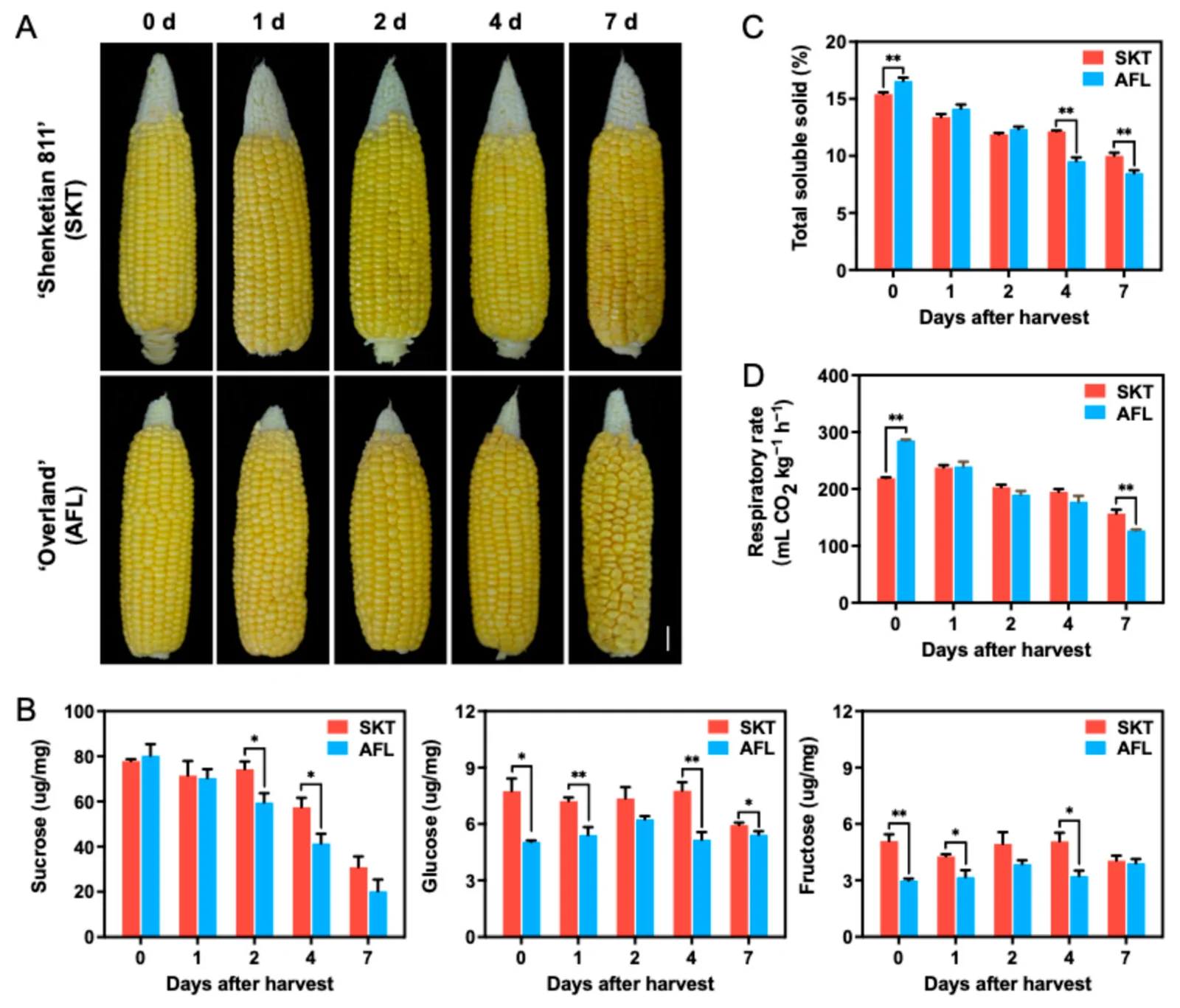
Changes in kernel appearance, soluble sugar contents, total soluble solids, and respiration rates during ambient storage of two sweet corn cultivars. (**A**) Representative ears of SKT and AFL at days 0, 1, 2, 4, and 7 after harvest during ambient storage. Bars = 2 cm; (**B**) sucrose, glucose, and fructose contents in kernels; (**C**) total soluble solids (TSSs); (**D**) respiration rates. For (**B**,**C**), error bars represent the standard error from three biological replicates. For (**D**), error bars represent the standard error from six biological replicates. Asterisks indicate significant differences between cultivars at the respective time points, determined by Student’s *t*-test (*, *p* < 0.05; **, *p* < 0.01).

Representative ears of the two sweet corn cultivars ‘SKT’ and ‘AFL’ exhibited progressive visual deterioration during ambient storage, with more pronounced kernel shriveling in AFL at day 7 (Figure 1A). Sucrose was the most abundant soluble sugar and showed the largest absolute change during storage in both cultivars (Figure 1B). Sucrose concentration decreased from 77.9 μg mg^−1^ fresh weight (FW) at harvest to 30.9 μg mg^−1^ at day 7 in SKT and from 80.3 to 20.3 μg mg^−1^ in AFL. Sucrose concentrations were comparable between cultivars at days 0 and 1. From day 2 onward, however, sucrose concentration was higher in SKT than in AFL, with significant differences observed at day 2 (74.3 versus 59.6 μg mg^−1^, *p* < 0.05) and day 4 (57.4 versus 41.4 μg mg^−1^, *p* < 0.05). Glucose and fructose concentrations were generally lower than sucrose concentrations. Glucose concentration was significantly higher in SKT than in AFL at days 0, 1, 4, and 7, whereas fructose concentration was significantly higher in SKT at days 0, 1, and 4. As sucrose depletion represented the most pronounced change in soluble sugar composition during ambient storage and is directly linked to eating quality, it was selected as the primary trait for subsequent transcriptomic analyses.

Total soluble solids (TSSs), an integrated indicator of soluble constituents in kernels, declined during storage in both cultivars (Figure 1C). AFL had a significantly higher TSS concentration than SKT at harvest (16.6% versus 15.4%, *p* < 0.01), whereas the ranking of the two cultivars was reversed at days 4 and 7. At the final sampling point, TSS concentration was 10.0% in SKT and 8.5% in AFL (*p* < 0.01). Cultivar differences in whole-ear respiration rate were mainly observed at the beginning and end of storage (Figure 1D). At harvest, AFL had a significantly higher respiration rate than SKT (285 versus 219 mL CO_2_ kg^−1^ h^−1^, *p* < 0.01); no significant difference was detected at days 1, 2, or 4. At day 7, the respiration rate was higher in SKT than in AFL (157 versus 127 mL CO_2_ kg^−1^ h^−1^, *p* < 0.01).

### 2.2. Transcriptome Quality and Weighted Gene Co-Expression Network Analysis

To characterize transcriptional responses associated with the distinct sucrose trajectories, kernels of SKT and AFL were subjected to RNA sequencing at 0, 1, 2, 4, and 7 days after harvest. Three biological replicates were included for each cultivar and time combination, generating 30 libraries in total. The dataset yielded 198 Gb of clean bases, with 5.98–7.31 Gb per library. Q30 values ranged from 96.2% to 96.8%, GC contents from 52.4% to 55.6%, and 89.0–91.9% of clean reads mapped to the maize B73 reference genome version 5 (Table S1).

**Figure 2:**
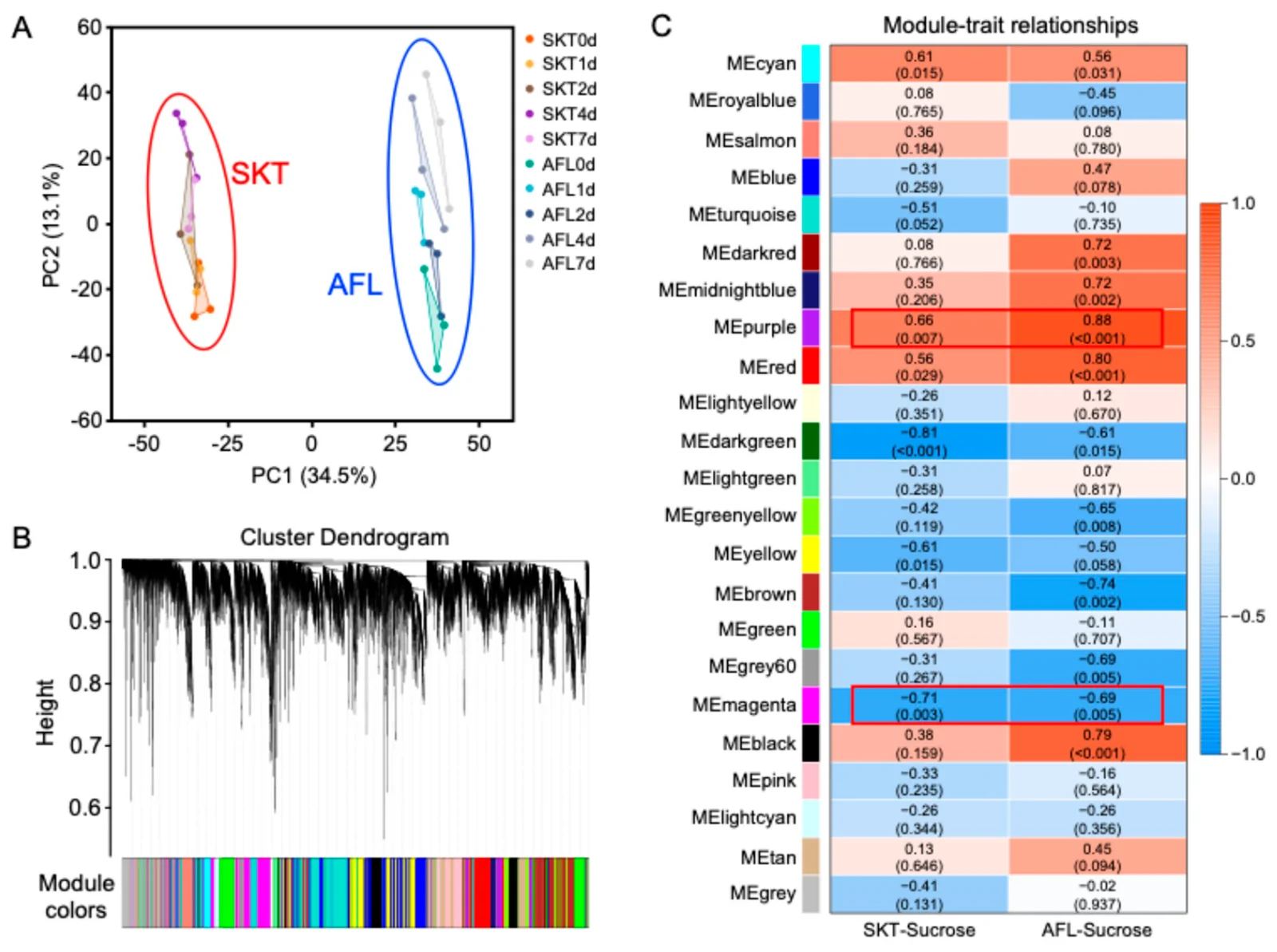
Principal component analysis (PCA) and weighted gene co-expression network analysis (WGCNA) of kernel transcriptomes during ambient storage. (**A**) PCA of 30 RNA-seq libraries from SKT and AFL kernels collected at days 0, 1, 2, 4, and 7 after harvest. The red and blue circles outline the 15 libraries of SKT and AFL, respectively; (**B**) hierarchical clustering dendrogram of expressed genes and the corresponding WGCNA module assignments; (**C**) relationships between module eigengenes and kernel sucrose content. Values in cells indicate Pearson correlation coefficients, with *p* values shown in parentheses. Red boxes highlight the MEpurple and MEmagenta modules, which show correlations with sucrose content in the same direction in both cultivars, with |*r*| > 0.65 and *p* < 0.01.

Principal component analysis (PCA) separated SKT and AFL primarily along PC1, which explained 34.5% of the total variance (Figure 2A). PC2 explained an additional 13.1% and partially separated samples by storage time. Biological replicates from the same cultivar and time combination generally clustered together. These results indicated that cultivar background was a major source of transcriptional variation, with an additional contribution from storage time.

Weighted gene co-expression network analysis (WGCNA) was performed using expressed genes from the 30 libraries. A soft-thresholding power of 11 was selected to approximate scale-free topology (Figure S1), yielding 23 co-expression modules (Figure 2B). To assess whether module–sucrose associations were reproduced within each cultivar, Pearson correlations between module eigengenes and sucrose concentration were calculated separately for SKT and AFL. Modules were retained when they showed correlations in the same direction in both cultivars, with |*r*| > 0.65 and *p* < 0.01 in each cultivar. Only MEpurple and MEmagenta met these criteria (Figure 2C). MEpurple was positively correlated with sucrose concentration in SKT (*r* = 0.66, *p* = 0.007) and AFL (*r* = 0.88, *p* < 0.001), whereas MEmagenta was negatively correlated with sucrose concentration in SKT (*r* = −0.71, *p* = 0.003) and AFL (*r* = −0.69, *p* = 0.005). MEpurple and MEmagenta contained 434 and 637 genes, respectively. Together, the 1071 genes in these two modules were subjected to subsequent gene-level correlation analysis.

### 2.3. Identification and Functional Enrichment of Sucrose-Associated Genes

**Figure 3:**
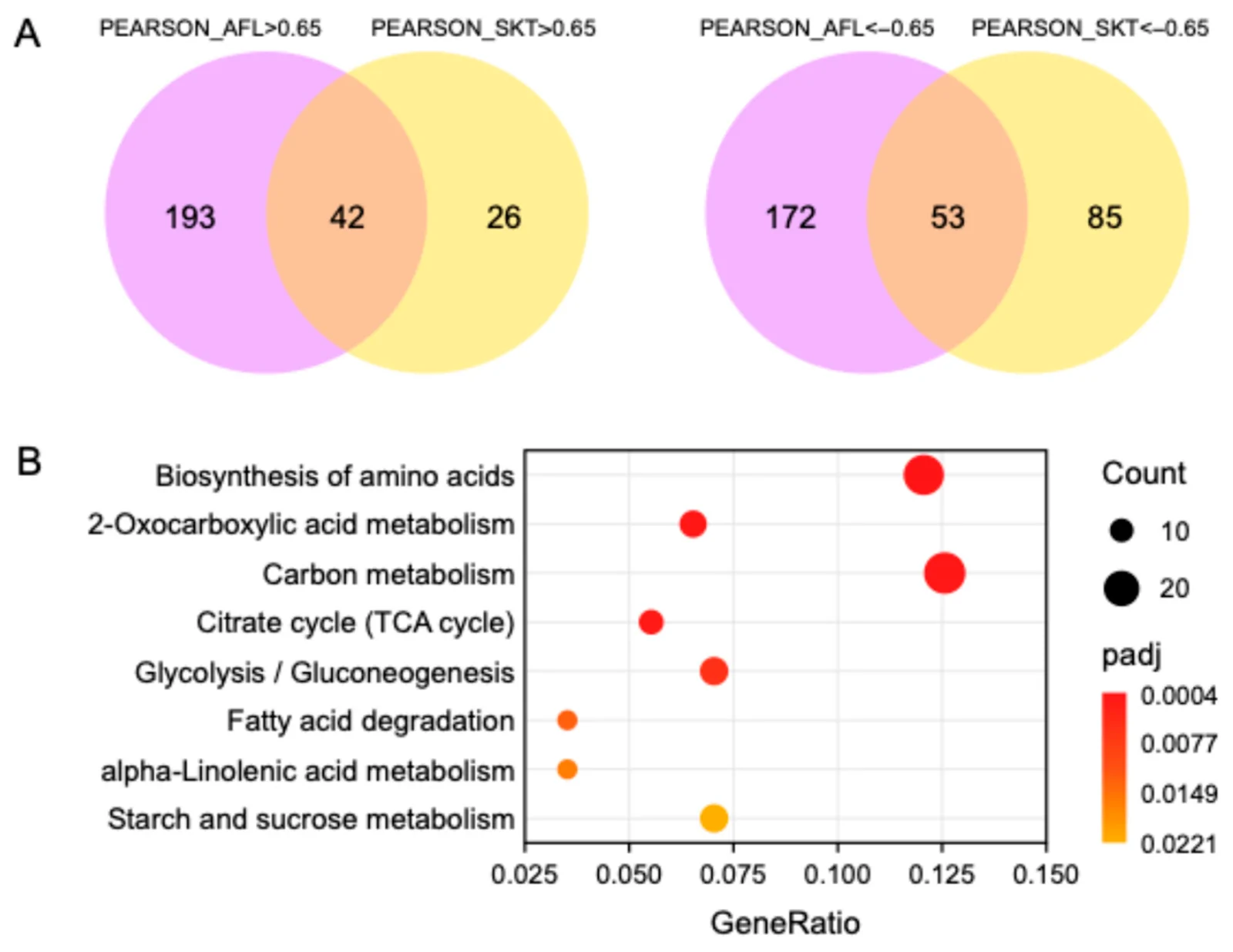
Identification of cultivar-specific sucrose-associated genes and functional enrichment analysis. (**A**) Venn diagrams showing genes whose transcript abundance was positively or negatively correlated with sucrose content within AFL and SKT (|*r*| > 0.65); (**B**) KEGG enrichment analysis of the 571 non-redundant sucrose-associated genes, showing the significantly enriched pathways (adjusted *p* ≤ 0.05).

Pearson correlations between transcript abundance and sucrose concentration were calculated separately for the 1071 genes using the 15 samples from each cultivar. Genes with |*r*| > 0.65 in either cultivar were retained as sucrose-associated genes. Among the positively correlated genes, 235 and 68 met the threshold in AFL and SKT, respectively, with 42 shared between the cultivars. Among the negatively correlated genes, 225 and 138 met the threshold in AFL and SKT, respectively, with 53 shared between the cultivars (Figure 3A). This notably higher number of sucrose-responsive transcripts in AFL was consistent with its more severe metabolic deterioration observed during ambient storage. After removing overlaps, 571 non-redundant sucrose-associated genes were identified.

KEGG enrichment analysis of the 571 genes identified eight significantly enriched pathways (adjusted *p* ≤ 0.05; Figure 3B), whereas the remaining pathways did not reach the significance threshold (Table S2). Biosynthesis of amino acids showed the strongest enrichment (24 genes, adjusted *p* = 4.29 × 10^−4^), followed by 2-oxocarboxylic acid metabolism (13 genes, adjusted *p* = 7.90 × 10^−4^) and carbon metabolism (25 genes, adjusted *p* = 8.27 × 10^−4^). The citrate cycle, glycolysis/gluconeogenesis, fatty acid degradation, α-linolenic acid metabolism, and starch and sucrose metabolism were also significantly enriched. These results indicated that the sucrose-associated genes encompassed starch and sucrose metabolism as well as broader pathways involved in postharvest carbon and energy metabolism.

### 2.4. Identification of Storage-Responsive and Cultivar-Dependent Carbon Metabolism Candidate Genes

**Figure 4:**
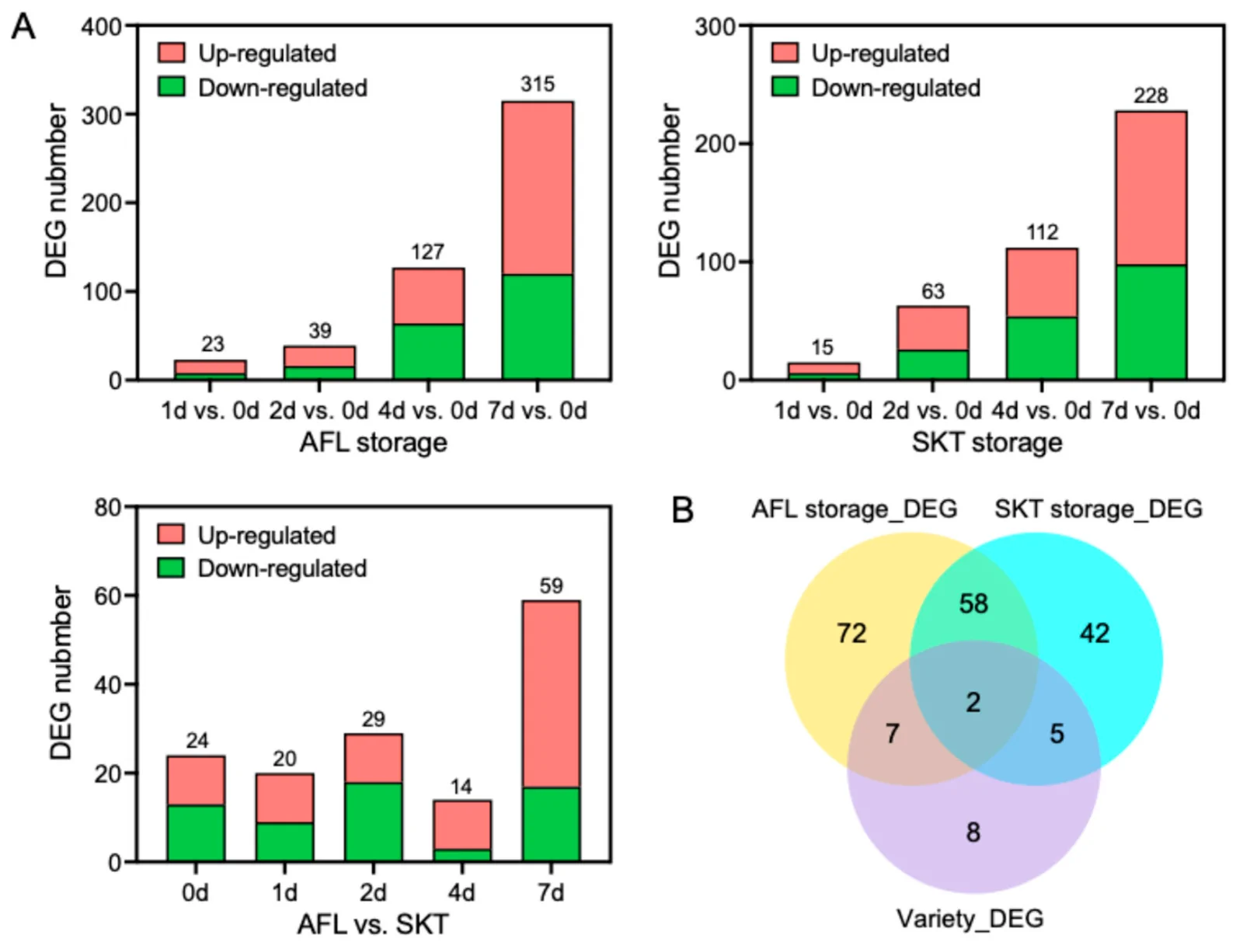
Differential expression analysis identifies storage-responsive and cultivar-dependent candidate genes. (**A**) Numbers of up- and down-regulated differentially expressed genes (DEGs) in AFL and SKT during storage, based on comparisons of each storage time with day 0, and in AFL-versus-SKT comparisons at each storage time. Numbers above bars indicate total DEGs; (**B**) overlap among recurrent AFL storage-responsive DEGs, SKT storage-responsive DEGs, and cultivar-differential DEGs. Within each category, genes exhibiting differential expression in the same direction in at least two comparisons were retained.

To prioritize genes showing recurrent responses to storage or consistent differences between cultivars, the 571 sucrose-associated genes were subjected to pairwise differential-expression analyses. Within each cultivar, transcript abundance at days 1, 2, 4, and 7 was compared with that at harvest (day 0), and AFL and SKT were compared at each time point. DEGs were defined by |log_2_(fold change)| ≥ 1 and false discovery rate (FDR) ≤ 0.05. The number of storage-responsive DEGs increased over time in both cultivars. In AFL, 23, 39, 127, and 315 DEGs were identified at days 1, 2, 4, and 7 relative to day 0, respectively, compared with 15, 63, 112, and 228 DEGs in SKT. Comparisons between cultivars identified 24, 20, 29, 14, and 59 DEGs at days 0, 1, 2, 4, and 7, respectively (Figure 4A and Figure S2). Genes showing differential expression in the same direction in at least two comparisons within a contrast category were retained, yielding 139 storage-responsive genes in AFL, 107 in SKT, and 22 cultivar-dependent genes. After removing overlaps, 194 non-redundant genes remained (Figure 4B and Table S3).

Among the 194 genes, five annotated as participating in sucrose cleavage or sucrose–starch interconversion were prioritized (Table 1). Two cytoplasmic phosphoglucomutase genes (*Zm00001eb054980* and *Zm00001eb216260*) were positively correlated with sucrose concentration and recurrently downregulated during storage in AFL. Genes encoding UTP–glucose-1-phosphate uridylyltransferase (*Zm00001eb104400*) and 1,4-α-glucan branching enzyme (*Zm00001eb084160*) were positively correlated with sucrose concentration and recurrently downregulated in SKT. Genes encoding cell-wall invertase 1 (*ZmINVCW1*; *Zm00001eb242820*) was negatively correlated with sucrose concentration in SKT, recurrently upregulated during SKT storage, and expressed at higher levels in AFL than in SKT at multiple sampling times. As the only gene combining storage-responsive and cultivar-dependent expression with a direct role in sucrose hydrolysis, *ZmINVCW1* was selected for further analysis.

### 2.5. ZmINVCW1 Is Associated with Cultivar Differences in Sucrose Depletion

**Figure 5:**
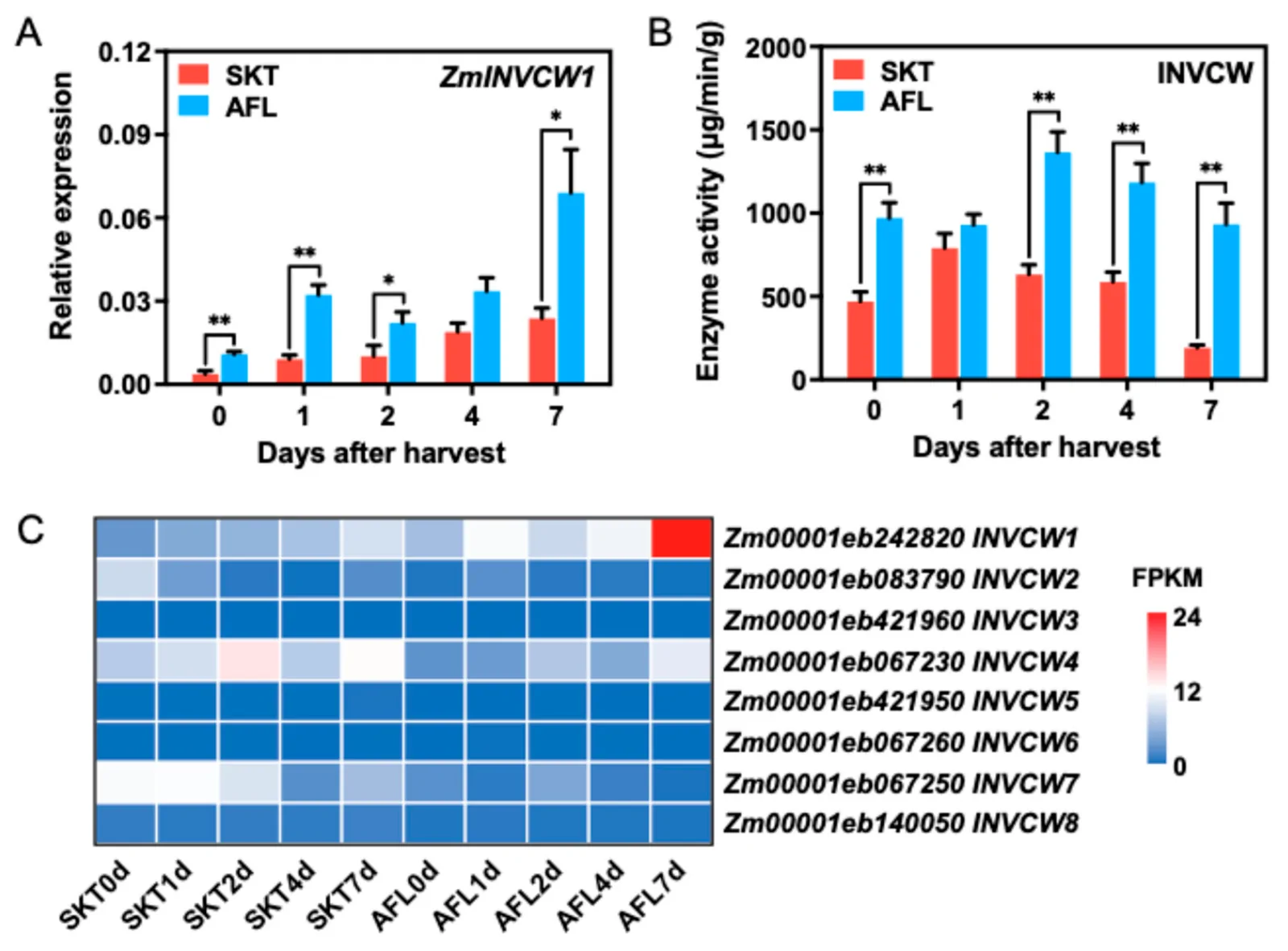
Expression profiles and enzymatic activities of cell wall invertases (INVCWs) during ambient storage. (**A**) Relative expression of *ZmINVCW1* determined by RT-qPCR in SKT and AFL kernels during ambient storage; (**B**) INVCW enzymatic activity in kernels of the two cultivars. For (**A**) and (**B**), error bars represent the standard error from three biological replicates, and asterisks indicate significant differences between cultivars using two-way ANOVA (*, *p* < 0.05; **, *p* < 0.01); (**C**) Heatmap showing FPKM values of eight *ZmINVCW* genes across cultivar and storage-time combinations.

Among the five prioritized genes, *ZmINVCW1* displayed several features associated with sucrose depletion and cultivar differences. In SKT, its transcript abundance was negatively correlated with sucrose concentration (*r* = −0.84) and increased in multiple storage contrasts (Table 1). It was also expressed at higher levels in AFL than in SKT at multiple sampling times. RT-qPCR confirmed the cultivar-dependent expression pattern detected by RNA-seq (Figure 5A), with significantly higher expression in AFL at days 0, 1, 2, and 7. Across the 30 matched samples, RNA-seq FPKM values were strongly correlated with RT-qPCR relative-expression values (Pearson’s *r* = 0.93, *p* < 0.001).

Total cell-wall invertase activity also differed between cultivars (Figure 5B). The enzyme activity was significantly higher in AFL than in SKT at days 0, 2, 4, and 7, consistent with the direction of the cultivar-differential *ZmINVCW1* expression. The temporal profiles of these measurements in SKT were not strictly synchronous: cell-wall invertase activity increased at day 1 and subsequently declined, whereas *ZmINVCW1* transcript abundance increased during storage (Figure 5A,B). As the measured cell-wall invertase activity reflects the combined activity of multiple INVCW isoenzymes, the activity differences could not be attributed specifically to *ZmINVCW1*. We therefore examined the expression profiles of eight curated *INVCW* genes identified according to the maize invertase-family classification [22] (Figure 5C). *INVCW2* and *INVCW7* were more highly expressed in SKT during early storage and subsequently decreased, whereas *INVCW4* maintained moderate to high expression in both cultivars; the remaining members showed low or undetectable expression. Among the eight genes, only *ZmINVCW1* showed continuously higher mean expression in AFL than in SKT at all five time points. These results support prioritizing *ZmINVCW1* as a candidate associated with cultivar differences in sucrose depletion; however, its contribution to total cell-wall invertase activity and sucrose loss requires functional validation.

## 3. Discussion

This study examined physiological and transcriptional differences associated with postharvest sucrose retention in SKT and AFL under ambient laboratory conditions. After 7 days of storage, sucrose concentrations decreased by approximately 60% in SKT and 75% in AFL relative to harvest. SKT also retained higher TSSs during the later storage period and exhibited less kernel shriveling, indicating better retention of organoleptic quality and appearance. Cultivar-dependent differences in postharvest sucrose retention and sugar-metabolic responses have also been observed during storage at 20 °C [17]. Using a recombinant inbred population derived from two supersweet corn parents with contrasting rates of postharvest sugar loss, Ren et al. [20] mapped soluble-sugar degradation to multiple regions on chromosomes 2, 4, and 10, supporting its quantitative genetic basis. The present study extends these observations by relating cultivar-dependent sucrose loss to changes in gene expression and cell-wall invertase activity.

Principal component analysis separated the samples primarily by cultivar, indicating that genetic background was a major source of transcriptional variation. As module–trait correlations were calculated from 15 libraries per cultivar, statistical power for detecting moderate associations was limited. Module prioritization was therefore based on strong correlations in the same direction in both cultivars. The integrated WGCNA, gene–sucrose correlation, and differential-expression analyses identified recurrently responsive genes associated with carbohydrate metabolism. Among these, *ZmINVCW1* generally showed higher transcript abundance in AFL than in SKT, and RT-qPCR confirmed its differential expression at multiple storage times. Total cell-wall invertase (INVCW) activity was also higher in AFL at days 0, 2, 4, and 7. Notably, the higher INVCW activity in AFL at days 2 and 4 occurred when its sucrose content was significantly lower than that of SKT. However, the temporal profiles of *ZmINVCW1* expression and total INVCW activity were not fully concordant. In SKT, *ZmINVCW1* transcript abundance increased during storage, whereas total INVCW activity peaked at day 1 and subsequently declined. Moreover, the negative correlation between *ZmINVCW1* expression and sucrose content was significant in SKT but not in AFL, suggesting that the strength of this relationship depends on the cultivar background. The significant temporal association in SKT, together with the consistently higher transcript abundance in AFL, supports the prioritization of *ZmINVCW1* as a candidate associated with cultivar-dependent sucrose retention. The enzyme-activity results independently implicate cell-wall invertase metabolism in the observed sucrose changes. However, as the assay measured the combined activity of multiple cell-wall invertase isoforms, the contribution of *ZmINVCW1* cannot be quantified from the present data. Post-transcriptional regulation, protein turnover, other INVCW family members, and additional sucrose-cleaving enzymes may contribute to the differences between transcript abundance and total enzyme activity.

Previous functional studies provide an important context for interpreting the INVCW family. Taliercio et al. [23] identified *Incw1* and *Incw2* as two maize cell-wall invertase genes and demonstrated marked differences in their temporal and tissue-specific expression. Cheng et al. [24] subsequently established that *Miniature1* (*Mn1*) encodes INCW2. INCW2 is predominantly expressed in basal endosperm transfer cells during early kernel development, where it contributes to sucrose unloading, sink strength, and normal kernel development [25,26]. The residual cell-wall invertase activity in *mn1* mutant kernels has been attributed to *Incw1*, which was expressed during the storage-product accumulation phase of endosperm development [25]. In the present study, *ZmINVCW1* generally showed higher expression in AFL across the five sampling times, whereas *ZmINVCW2* did not exhibit sustained cultivar-biased expression. This expression pattern supported the prioritization of *ZmINVCW1* as a cultivar-associated candidate, but gene-specific functional analyses are required to determine its relative contribution during postharvest storage.

Previous postharvest studies also indicate that invertase participates in a broader sugar-metabolism network. Chen et al. [17] found that ‘Nongkenuo 336’, which showed the smallest decrease in sucrose during storage at 20 °C, exhibited smaller increases in *INV* and *BMY* expression and smaller decreases in *SS4* and *SS5* expression than the other two sweet corn cultivars. Liu et al. [27] found that ClO_2_ treatment delayed the decrease in TSSs while suppressing the expression of *IN*, *FRK*, and *GPI*. Improved sugar retention at 0 and 4 °C was similarly accompanied by lower expression of *IN*, *FK*, *HXK*, and *PFK* and higher expression of sucrose-synthesis-related genes, including *SPP*, *SPS*, and *SUS* [28]. Li et al. [29] also reported that ozone treatment delayed sucrose decline, accompanied by higher *SUS* and *SPS* expression and lower *IN* expression. Although these studies examined environmental treatments rather than intrinsic cultivar differences, they consistently associate postharvest sugar retention with coordinated changes in invertase and other sugar-metabolism genes. Analysis of an additional dataset from the sweet corn cultivar ‘Hetianyu 818’ showed that *ZmINVCW1* expression increased during storage at 25 °C, whereas this induction was attenuated at 5 °C. Across the two temperature conditions, its expression was significantly negatively correlated with sucrose content (Figure S3). These results provide independent support for the negative association between *ZmINVCW1* expression and sucrose content, and further indicate that its postharvest expression response is influenced by storage temperature.

Glucose and fructose concentrations did not closely parallel sucrose decline, leaving the fate of sucrose-derived carbon unresolved. A more complete assessment of postharvest carbon partitioning will require measurements of sucrose synthase, sucrose-phosphate synthase, other invertases, and starch content. The specific contribution of *ZmINVCW1* should be evaluated using isoform-specific protein quantification and enzyme assays, analyses of alternative splicing, tissue-specific expression, subcellular localization, and functional validation through knockout, overexpression, and genetic complementation. Taken together, these findings prioritize *ZmINVCW1* as a candidate associated with cultivar-dependent postharvest sucrose loss and provide a foundation for investigating its functional role.

## 4. Materials and Methods

### 4.1. Plant Materials and Storage Conditions

Two commercial sweet corn hybrids, ‘Shenketian 811’ (SKT) and ‘Overland’ (AFL), were used in this study. Both hybrids are popular among consumers in Shanghai, China, and are representative *sh2*-type supersweet corn hybrids carrying the recessive *shrunken2* (*sh2*) allele. The plants were grown in spring 2025 at the Zhuanghang Experimental Station of the Shanghai Academy of Agricultural Sciences, Shanghai, China (30°53′ N, 121°23′ E). They were planted in double rows with a row spacing of 0.60 m and an interplant spacing of 0.25 m and managed according to traditional field management practices.

Ears of both hybrids were harvested concurrently at 20 days after pollination, corresponding to the commercial milk stage. Uniform ears without visible mechanical damage, disease symptoms, or developmental defects were selected and immediately transported to the laboratory. Intact ears with husks were stored at ambient temperature (24–26 °C) with 60–70% relative humidity. Samples were collected at harvest (0 d) and after 1, 2, 4, and 7 days of storage. For each hybrid at each sampling time, kernels from the middle third of three ears were pooled as one biological replicate, and three independent biological replicates were prepared. Samples intended for biochemical and molecular analyses were immediately frozen in liquid nitrogen and stored at −80 °C until use.

### 4.2. Respiration-Rate Measurement

The respiration rate was determined using a Dansensor^®^ CheckPoint3 portable headspace gas analyzer (MOCON Europe A/S, Ringsted, Denmark) [30]. Husks were removed immediately before measurement to exclude CO_2_ production by the husk tissues and minimize variation associated with differences in husk mass and coverage. Individual ears were weighed and sealed separately in a 2.25 L airtight chamber. The headspace CO_2_ concentration was recorded immediately after sealing (*C*_0_) and after 1 h (*C_t_*). The effective headspace volume was calculated by subtracting the estimated ear volume from the chamber volume. Ear volume was approximated from fresh weight by assuming an ear density of 1.0 kg L^−1^. The respiration rate was calculated as follows: *R* = [(*C_t_* − *C*_0_) × (*V_c_* − *m*/*ρ*) × 1000]/[100 × *m* × *t*], where *R* is the respiration rate (mL CO_2_ kg^−1^ h^−1^), *C*_0_ and *C_t_* are the initial and final CO_2_ concentrations (%), respectively, *V_c_* is the chamber volume (2.25 L), *m* is the fresh weight of the ear (kg), *ρ* is the assumed ear density (1.0 kg L^−1^), and *t* is the enclosure time (1 h). For each cultivar and sampling time, six individual ears were analyzed as six independent biological replicates. Each ear was measured once, and all data were retained for statistical analysis.

### 4.3. Determination of Total Soluble Solids

Kernel samples were homogenized, and the resulting juice was filtered through cheesecloth. Total soluble solids (TSSs) were measured using a WYA-ZT automatic thermostatic Abbe refractometer (Shanghai INESA Physico-Optical Instrument Co., Ltd., Shanghai, China) and expressed as °Brix [31]. For each cultivar and sampling time, three independent biological replicates were analyzed, each comprising pooled kernels from three ears. One refractometric measurement was performed per biological replicate, and all data were retained for statistical analysis.

### 4.4. Determination of Soluble Sugars

Soluble sugars were extracted according to the method of Li et al. [32] with minor modifications. Fifty milligrams of frozen kernel powder was suspended in 700 μL of 80% (*v*/*v*) ethanol and extracted with continuous agitation at 50 °C for 120 min. After adding an equal volume (700 μL) of ultrapure water, the suspension was centrifuged at 10,000 rpm for 3 min. The clarified extract was collected and diluted appropriately. Glucose, fructose, and sucrose were quantified using a Dionex ICS-5000+ system fitted with a CarboPac^TM^ PA1 analytical column (4.0 × 250 mm; Thermo Fisher Scientific, Sunnyvale, CA, USA). The column was maintained at 30 °C, and 10 μL of each extract was injected. Sugar components were assigned based on standard retention times and quantified using external calibration. Sugar contents were expressed as μg mg^−1^ fresh weight. For each cultivar and sampling time, three independent biological replicates were analyzed, each comprising pooled kernels from the middle third of three ears. One chromatographic determination was performed per biological replicate, and all data were retained for statistical analysis.

### 4.5. RNA Sequencing and Differential-Expression Analysis

Kernel samples collected at 0, 1, 2, 4, and 7 days of storage from both cultivars were used for RNA sequencing, with three biological replicates per cultivar and time-point. Total RNA was isolated with the FastPure Universal Plant Total RNA Isolation Kit (Vazyme Biotech Co., Ltd., Nanjing, China) according to the manufacturer’s instructions. RNA concentration and purity were assessed using a NanoDrop spectrophotometer (Thermo Fisher Scientific, Waltham, MA, USA), and RNA integrity was evaluated by 1% agarose-gel electrophoresis. RNA libraries were constructed and sequenced on the Illumina platform by Novogene Co., Ltd. (Beijing, China). Adapter, poly-N, and low-quality reads were removed using Trimmomatic v0.36 [33]. Clean reads were mapped to the *Zea mays* B73 RefGen_v5 genome assembly (NCBI accession GCF_902167145.1) using HISAT2 v2.0.5 [34]. Transcript abundance was expressed as fragments per kilobase of transcript per million mapped fragments (FPKM) for expression-profile visualization. Differential expression was assessed from the raw count matrix using DESeq2 v1.20.0 [35]. Within each cultivar, samples collected at 1, 2, 4, and 7 days were compared with those collected at 0 day. AFL and SKT were also compared at each storage time point. Genes with |log_2_(fold change)| ≥ 1 and FDR ≤ 0.05 were considered DEGs. Principal component analysis and KEGG enrichment analysis were performed using the NovoMagic platform (https://magic-plus.novogene.com (accessed on 1 August 2026)).

### 4.6. Weighted Gene Co-Expression Network and Candidate-Gene Analysis

Weighted gene co-expression network analysis (WGCNA) was conducted using the WGCNA R package v1.74 [36]. A total of 19,895 genes with a mean FPKM ≥ 1 and a coefficient of variation > 0.4 across the 30 RNA-seq libraries were retained for network construction. A soft-thresholding power of 11 was selected to approximate scale-free topology. Modules with similar eigengene profiles were merged at a correlation threshold of 0.75, and the minimum module size was set to 30 genes. After network construction, Pearson’s correlations between module eigengenes and sucrose content were calculated separately within the 15 AFL and 15 SKT libraries. The modules showing the strongest concordant positive and negative correlations with sucrose in both cultivars were selected for subsequent analysis.

At the gene level, Pearson’s correlation coefficients between gene expression and sucrose content were also calculated separately within the 15 AFL and 15 SKT libraries. Genes with |*r*| > 0.65 in either cultivar were retained as sucrose-associated genes. These genes were subsequently examined in within-cultivar storage and between-cultivar DEG comparisons. Genes showing significant upregulation or downregulation in at least two storage comparisons, or significant differential expression in at least two AFL–SKT comparisons, were retained as recurrent storage-responsive or cultivar-dependent candidate genes.

### 4.7. RT-qPCR Analysis

Total RNA was reverse-transcribed using HiScript III RT SuperMix for qPCR with gDNA wiper (Vazyme Biotech Co., Ltd., Nanjing, China). Briefly, 1 μg of total RNA was used for cDNA synthesis, and the resulting cDNA was diluted tenfold before RT-qPCR analysis. RT-qPCR was performed using ChamQ Universal SYBR qPCR Master Mix (Vazyme Biotech Co., Ltd., Nanjing, China) on a LineGene 9600 Plus Real-Time PCR System (Bioer Technology Co., Ltd., Hangzhou, China). *ZmActin* was used as the reference gene [37]. Relative transcript abundance was calculated using the 2^−ΔCt^ method. Three independent biological replicates were analyzed for each cultivar and sampling time point. Primers were designed using Primer Premier 5, and their specificity was verified using NCBI Primer-BLAST (https://www.ncbi.nlm.nih.gov/tools/primer-blast (accessed on 1 August 2026)), melting-curve analysis and Sanger sequencing of the PCR products. Primer sequences are provided in Table S4.

### 4.8. Cell-Wall Invertase Activity Assay

Cell-wall-bound acid invertase (INVCW) activity was measured using a commercial assay kit (Jiangsu Aidisheng Biotechnology Co., Ltd., Yancheng, China) according to the manufacturer’s instructions. Briefly, approximately 0.1 g of frozen kernel tissue was homogenized on ice. The cell-wall fraction was collected by centrifugation, washed, and extracted overnight at 4 °C. INVCW activity was determined from the production of reducing sugars using the 3,5-dinitrosalicylic acid method, with absorbance measured at 540 nm using a SpectraMax 190 microplate reader (Molecular Devices, San Jose, CA, USA). Activity was expressed as μg min^−1^ g^−1^ fresh weight. Three biological replicates were analyzed for each cultivar and sampling time point.

### 4.9. Statistical Analysis

Statistical analyses and general data visualization were performed using GraphPad Prism 10 (GraphPad Software, LLC, Boston, MA, USA). Significant differences were calculated by Student’s *t*-test and two-way ANOVA. The heatmap and Venn diagram were generated using TBtools-II (South China Agricultural University, Guangzhou, China) [38].

## Figures and Tables

**Table 1:** Sucrose metabolism-associated candidate genes exhibiting recurrent storage-responsive or cultivar-dependent expression. Pearson’s correlation coefficients (*r*) between gene expression and sucrose content were calculated separately for AFL and SKT. Storage-responsive genes were upregulated (↑) or downregulated (↓) relative to day 0 in at least two storage comparisons. For cultivar-dependent genes, ‘AFL > SKT’ indicates significantly higher expression in AFL than in SKT at two or more sampling times. A dash (—) indicates that the gene did not meet the recurrent differential-expression criterion for the corresponding category.

Functional Category	Gene ID	Annotation	Sucrose Correlation and Storage Response		Cultivar Difference
			AFL	SKT	
Sucrose cleavage	*Zm00001eb242820*	Cell-wall invertase 1 (ZmINVCW1)	−0.48; —	−0.84; ↑	AFL > SKT
Hexose–phosphate interconversion	*Zm00001eb054980*	Phosphoglucomutase	0.86; ↓	0.73; —	—
Hexose–phosphate interconversion	*Zm00001eb216260*	Phosphoglucomutase	0.86; ↓	0.68; —	—
UDP-glucose formation	*Zm00001eb104400*	UTP–glucose-1-phosphate uridylyltransferase	0.82; —	0.70; ↓	—
Starch biosynthesis	*Zm00001eb084160*	1,4-α-glucan branching enzyme	0.87; —	0.67; ↓	—

## Data Availability

Transcriptome data (accession number CRA051351) are available at National Genomics Data Center, China National Center for Bioinformation.

## Supplementary Materials

The following supporting information can be downloaded at: https://www.mdpi.com/article/10.3390/plants15213413/s1, Figure S1: Selection of the soft-thresholding power for weighted gene co-expression network analysis (WGCNA); Figure S2: Overlap of pairwise differential-expression results for the 571 sucrose-associated genes during postharvest storage and between cultivars; Figure S3: Expression of *ZmINVCW1* and its relationship with sucrose content in sweet corn cultivar ‘Hetianyu 818’ during storage at 5 and 25 °C; Table S1: Summary of RNA-sequencing quality for kernel samples of AFL and SKT during postharvest storage; Table S2: Results of KEGG pathway enrichment analysis; Table S3: List of 194 sucrose-associated candidate genes exhibiting significant differential expression during storage or between cultivars; Table S4: List of primers used for RT-qPCR validation.
