# Supplementary material for: Time-Resolved Transcriptomics Provides Insights into Cultivar-Dependent Sucrose Loss During Ambient Storage of Sweet Corn
Source: Plants (Basel). 2026 Nov 3;15(21):3413. doi: 10.3390/plants15213413 (PMC13766073; doi:10.3390/plants15213413)
Supplement: Supplementary file 1 [file plants-15-03413-s001.zip › Supplementary Figures.pdf]

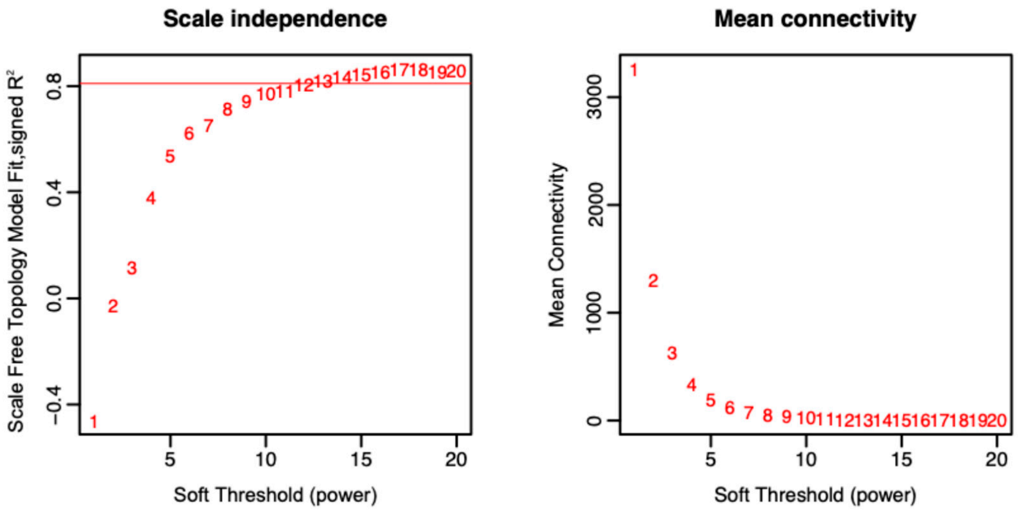

Supplementary Figure S1 Selection of the soft-thresholding power for weighted gene co-expression network analysis (WGCNA).

Scale-free topology fit index and mean connectivity across candidate soft-thresholding powers. A power of 11 was selected for network construction.

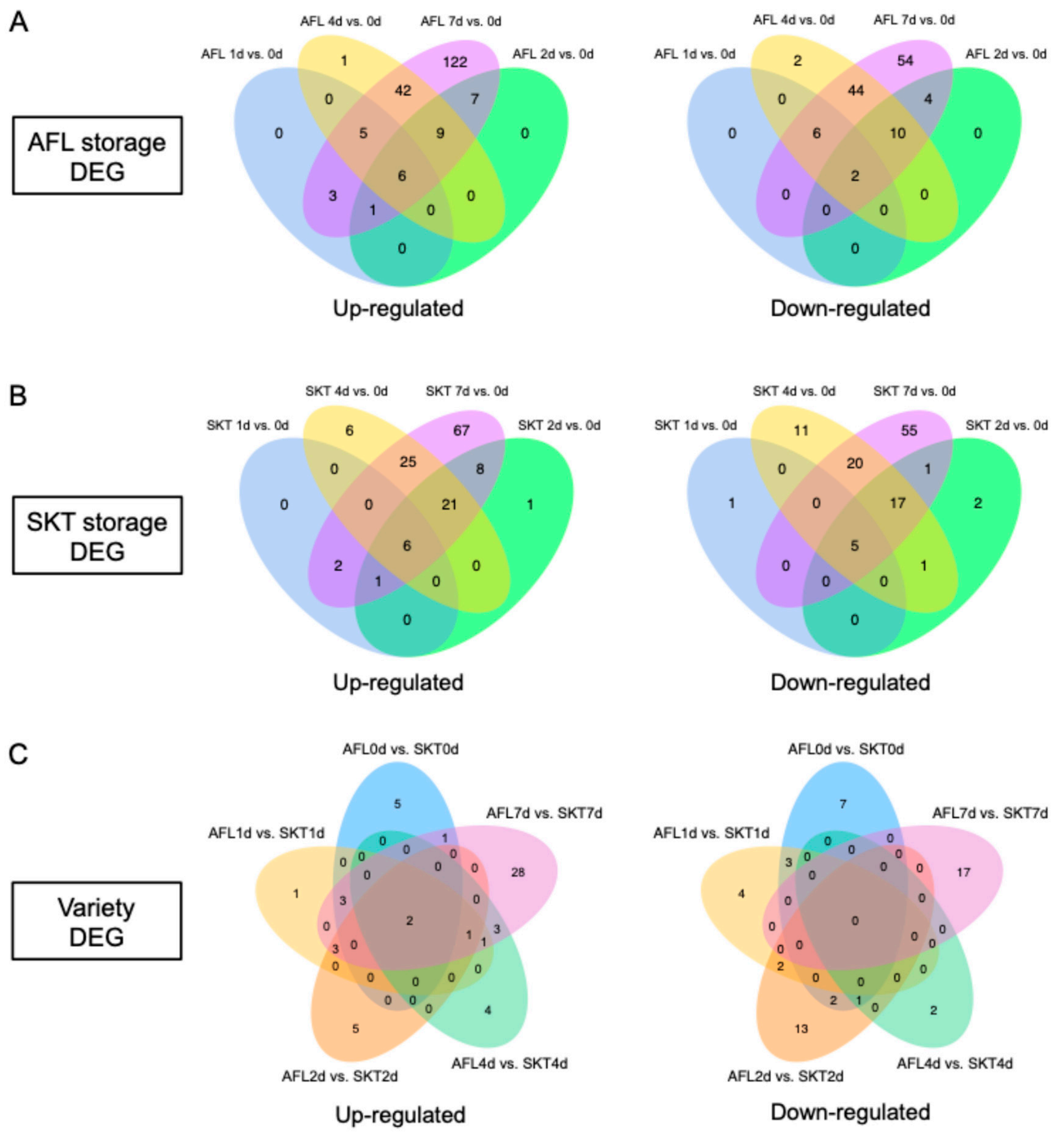

Supplementary Figure S2 Overlap of pairwise differential-expression results for the 571 sucrose-associated genes during postharvest storage and between cultivars.

(A) Venn diagram showing the overlap of differentially expressed genes (DEGs) for AFL at 1, 2, 4, and 7 d relative to 0 d; (B) Venn diagram showing the overlap of DEGs for SKT at 1, 2, 4, and 7 d relative to 0 d; (C) Venn diagram showing the overlap of DEGs for AFL–SKT comparisons at 0, 1, 2, 4 and 7 days of storage.

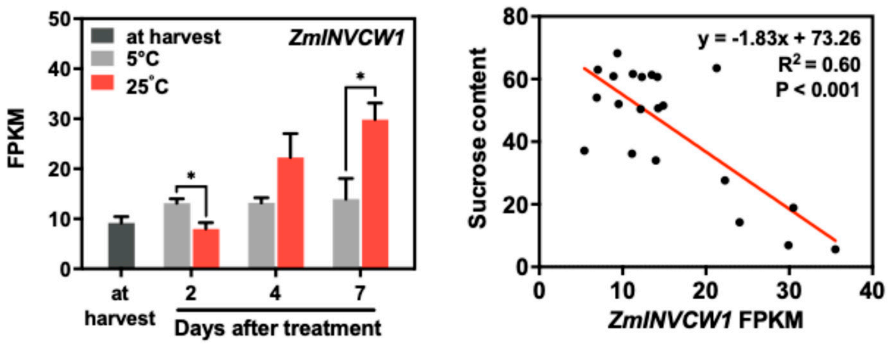

Supplementary Figure S3 Expression of *ZmINVCW1* and its relationship with sucrose content in sweet corn cultivar 'Hetianyu 818' during storage at 5 and 25 °C.

Error bars represent the standard error from three biological replicates. Asterisks indicate significant differences between cultivars using Student's *t*-test (*, $P < 0.05$).
